# *Dermacentor reticulatus* ticks (Acari: Ixodidae) distribution in north-eastern Poland: an endemic area of tick-borne diseases

**DOI:** 10.1007/s10493-018-0274-7

**Published:** 2018-07-19

**Authors:** Katarzyna Kubiak, Hanna Sielawa, Janina Dziekońska-Rynko, Dariusz Kubiak, Martyna Rydzewska, Ewa Dzika

**Affiliations:** 1 Department of Medical Biology, Faculty of Health Sciences, University of Warmia and Mazury in Olsztyn, Zolnierska 14c, 10-561 Olsztyn, Poland; 2 Department of Zoology, Faculty of Biology and Biotechnology, University of Warmia and Mazury in Olsztyn, Oczapowskiego 2, 10-719 Olsztyn, Poland; 3 Department of Microbiology and Mycology, Faculty of Biology and Biotechnology, University of Warmia and Mazury in Olsztyn, Oczapowskiego 2, 10-719 Olsztyn, Poland

**Keywords:** *Dermacentor reticulatus*, TICKS, Geographical distribution, Habitats, Poland

## Abstract

*Dermacentor reticulatus* is the second most important tick species in Poland. Although the north-eastern region of Poland is considered typical for *D. reticulatus* and is treated as a contiguous area of Eastern populations of the meadow ticks, the occurrence of this tick species in this region to date has been recorded as separate foci. The present report supplements data on the geographical distribution of *D. reticulatus* in urban and natural biotopes of north-eastern Poland (Warmia and Mazury province). In 2015–2017 (during the springtime activity of ticks) adult questing *D. reticulatus* were found in 13 of 25 monitored localities. Six sites are located in urbanized areas, within the administrative borders of the city of Olsztyn and seven sites are in natural biotopes in the central part of Warmia and Mazury. A total of 398 adult *D. reticulatus* ticks, including 257 females and 141 males, were collected. A comparison of data grouped according to urban and natural type of area revealed no statistical differences between them. Taking into account the habitat type, the mean tick density was the highest in open landscapes. The identification of new foci *D. reticulatus* in the endemic areas of Lyme borreliosis, tick-borne encephalitis and canine babesiosis is crucial for determining the risk of diseases transmitted by ticks and taking proper preventive measures.

## Introduction

The meadow tick *D. reticulatus* (Fabricius, 1974) is the second most abundant tick species in many parts of Europe after *Ixodes ricinus*. Due to the high adaptability of *D. reticulatus* to changing environmental conditions and a wide range of hosts, including livestock, domestic animals and humans, the veterinary-medical importance of this species has increased within in the last 20 years. Pathogens that can be transmitted to the host by *D. reticulatus* include the protozoa *Babesia canis*, bacteria of the genera *Rickettsia* and *Anaplasma,* and tick-borne encephalitis virus (Földvári et al. 2016). The significance of *D. reticulatus* in the transmission of *Borrelia burgdorferi* s.l. is still unclear, although the specific DNA of this pathogen has been detected in meadow ticks (Reye et al. 2013; Mierzejewska et al. 2015b; Kubiak et al. 2017; Zając et al. 2017), but this does not prove its role as a vector.

The geographical distribution of *D. reticulatus* in Europe ranges from northern Portugal, the western border of France and England through the countries of central Europe to Ukraine and to the east of Kazakhstan (Rubel et al. 2016). Until the 1980s the range of *D. reticulatus* was clearly divided between West-European and Eastern populations with an area from the Baltic Sea coast through central Germany, western Poland to the southern border of Hungary (Karbowiak 2014) where the meadow tick had never been reported. Nowadays, *D. reticulatus* shows a tendency of expansion into new areas in Germany (Dautel et al. 2006), Poland (Karbowiak and Kiewra 2010; Nowak 2011; Kiewra and Czulowska 2013; Mierzejewska et al. 2016), Slovakia (Bullová et al. 2009), the Czech Republic (Široký et al. 2011), Lithuania, Latvia (Paulauskas et al. 2015) and Romania (Chitimia-Dobler 2015) and has also been observed in countries of western Europe in locations where it had not been reported previously (Jongejan et al. 2015). The spread of *D. reticulatus* is influenced by many factors including climate changes due to global warming, changes in the way of using green areas, which contributes to the increase in the number and diversity of hosts as well as the development of tourism and transport between countries (Karbowiak 2014; Mierzejewska et al. 2017; Kloch et al. 2017).

Long-term monitoring of *D. reticulatus* in Poland has shown that the north-eastern and eastern parts of the country (east of the Vistula River) are endemic for this tick species (Szymański 1986; Siuda 1993; Bogdaszewska 2004; Biaduń 2011; Bartosik et al. 2011; Karbowiak 2014; Mierzejewska et al. 2016). In contrast, the western area of Poland between the Oder River (western border of the country) and the Vistula River, until the 1990s was considered to be free of this tick species (Szymański 1986; Siuda 1993; Bartosik et al. 2011; Karbowiak 2014). However, within the last two decades, due to the detection of new numerous localities of *D. reticulatus*, this part of Poland has been considered as an expansion area (Kadulski and Izdebska 2009; Karbowiak and Kiewra 2010; Nowak 2011; Kiewra and Czulowska 2013; Mierzejewska et al. 2016). The low genetic polymorphism between *D. reticulatus* from eastern Poland (eastern population), and newly emerged foci in western Poland, as well as considerable differences from the nearby German population, show that the foci in western Poland originated from the eastern *D. reticulatus* population (Kloch et al. 2017).

The present report supplements the data on the geographical distribution of *D. reticulatus* in the urban and natural biotopes of north-eastern Poland (Warmia and Mazury). Identification of new foci of *D. reticulatus* in the endemic areas of Lyme borrelioses, tick-borne encephalitis (TBE) and canine babesiosis (NIH-PZH; Mierzejewska et al. 2015b) is crucial for determining the risk of diseases transmitted by ticks and taking proper preventive measures.

## Materials and methods

**Fig. 1:**
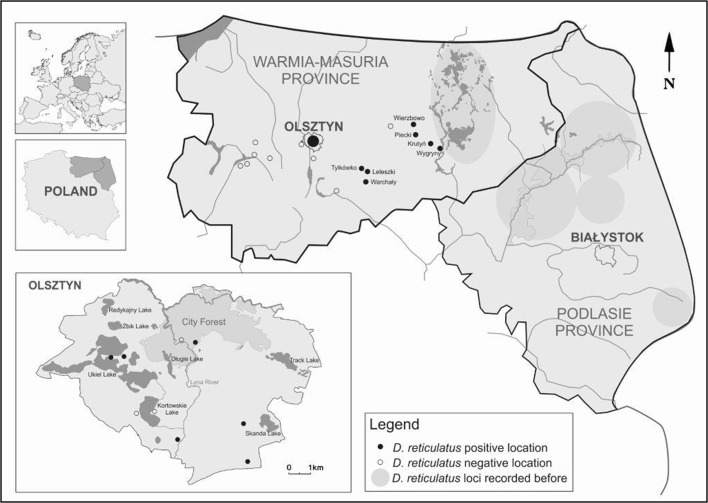
*Dermacentor reticulatus* positive and negative locations in north-eastern Poland

The occurrence of *D. reticulatus* was studied in 16 localities, across six districts of Warmia and Mazury from Szałkowo (N53°39′52.0″ E19°37′03.8″) in the west to Wygryny (N53°40′15.8″ E21°32′17.1″) in the east, in north-eastern Poland. Additionally, monitoring was carried out on the nine localities designated within the administrative boundaries of Olsztyn (88.0 km^2^), the capital of Warmia and Mazury (Table 1, Fig. 1).

**Table 1:** Localities and the number of questing adult *Dermacentor reticulatus* ticks collected in 2015–2017 in the Warmia and Mazury region, north-eastern Poland

Locality	Geographical coordinates	Habitats	Number of specimens								
			2015			2016			2017		
			F	M	T	F	M	T	F	M	T
Olsztyn-City Forest	53°47′51.9″N 20°28′35.0″E	Ecotone (meadow/forest)	7	3	10	18	7	25	14	7	21
Olsztyn-Brzeziny	53°44′46.9″N 20°27′34.8″E	Open landscape	7	8	15	10	8	18	45	22	67
Olsztyn-Mazurskie	53°45′39.4″N 20°31′06.1″E	Open landscape	12	10	22	39	36	75	15	3	18
Olsztyn-Teczowy Las	53°43′54.2″N 20°29′50.5″E	Ecotone (grassy path/forest)		nc		12	5	17	11	6	17
Olsztyn-Ukiel Lake (public watering area)	53°46′36.3″N 20°26′58.7″E	Ecotone (forested areas and lake shore vegetation		nc		7	1	8		nc	
Olsztyn-Ukiel Lake (Miła Bay)	53°46′23.9″N 20°26′25.1″E	Ecotone (forested areas and lake shore vegetation)		nc		2	0	2		nc	
Tylkówko	53^o^37′02.6 N 20^o^43′17.3E	Open landscapes		nc		16	10	26		nc	
Leleszki	53°37′31.9″N 20°49′51.7″E	Ecotone (grassy path/forest)		nc		14	4	18	3	0	3
Warchały (Barajnickie Lake)	53°32′15.6″N 20°49′14.6″E	Ecotone (forested areas and lake shore vegetation)		nc		4	1	5	2	0	2
Wierzbowo (close Mrągowo)	53°48′24.0″N 21°19′25.6″E	Ecotone (grassy path/forest)		nc		1	1	2	4	4	8
Piecki	53°48′01.7″N 21°19′33.1″E	Forest landscapes		nc		1	0	1	1	0	1
Krutyń	53°42′09.0″N 21°26′21.4″E	Forest landscapes		nc		2	1	3	1	0	1
Wygryny (close Ruciane Nida)	53°40′15.8″N 21°32′17.1″E	Ecotone (grassy path/forest)		nc		6	3	9	3	1	4

*F* females, *M* males, *T* total, *nc* no ticks collected

Tick sampling was conducted in the springtime activity of ticks (March–May) of 2015–2017 in different types of habitat: (a) in forest landscapes (mature mixed and deciduous forests and its borders), (b) in ecotones (zones between grassy and forested areas such as paths near forest borders, and forested areas and lake shore vegetation, (c) in open landscapes (meadows that are sparsely covered by trees or bushes; pastures), (d) in the urban landscape (city parks).

Ticks were collected from vegetation using the flagging method in the daytime between 9 a.m. and 3 p.m. by two persons for at least 30 min at each site. A site was qualified as positive if questing *D. reticulatus* were collected directly from vegetation, and was negative when none of the ticks were present on the blanket. Collected ticks were preserved in 70% ethanol and in the laboratory were identified to the species, sex and developmental stage using taxonomic keys (Siuda 1993; Nowak-Chmura 2013).

For a given location, the density of *D. reticulatus* ticks was estimated by determining the number of ticks per 100 m^2^ for each individual flagging event at a specific site. The differences in the abundance of ticks between urban and natural areas and in the different types of a habitat were estimated on the basis of the density of ticks collected in April 2016 (a month when all study localities were monitored). The data were analyzed using the non-parametric Mann–Whitney *U* test (*α* = 0.05). The test was conducted using the software package SPSS v.22.0 for Windows (SPSS, Chicago, IL, USA).

## Results

In north-eastern Poland, in 2015–2017, adult questing *D. reticulatus* were found in 13 of 25 monitored localities (Table 1, Fig. 1). Six sites are located in urbanized areas, within the administrative borders of the city of Olsztyn. The remaining seven sites are in natural biotopes in the central part of Warmia and Mazury. The localities where *D. reticulatus* was detected are in Szczytno (Tylkówko, Leleszki, Warchały), Mrągowo (Wierzbowo, Piecki, Krutyń) and Pisz (Wygryny) districts.

*D. reticulatus* ticks in Warmia and Mazury were collected in habitats typical for this species. Both in urban and non-urban areas, the habitats of *D. reticulatus* are ecotones—areas between forest and grassy paths or forest and lake shore vegetation or open landscapes such as meadows (Table 1). Only the Piecki and Krutyń localities are covered by mature deciduous trees. Ticks were not found in dense mature deciduous, coniferous and mixed forests or in the city park at Lake Kortowskie in Olsztyn.

**Fig. 2:**
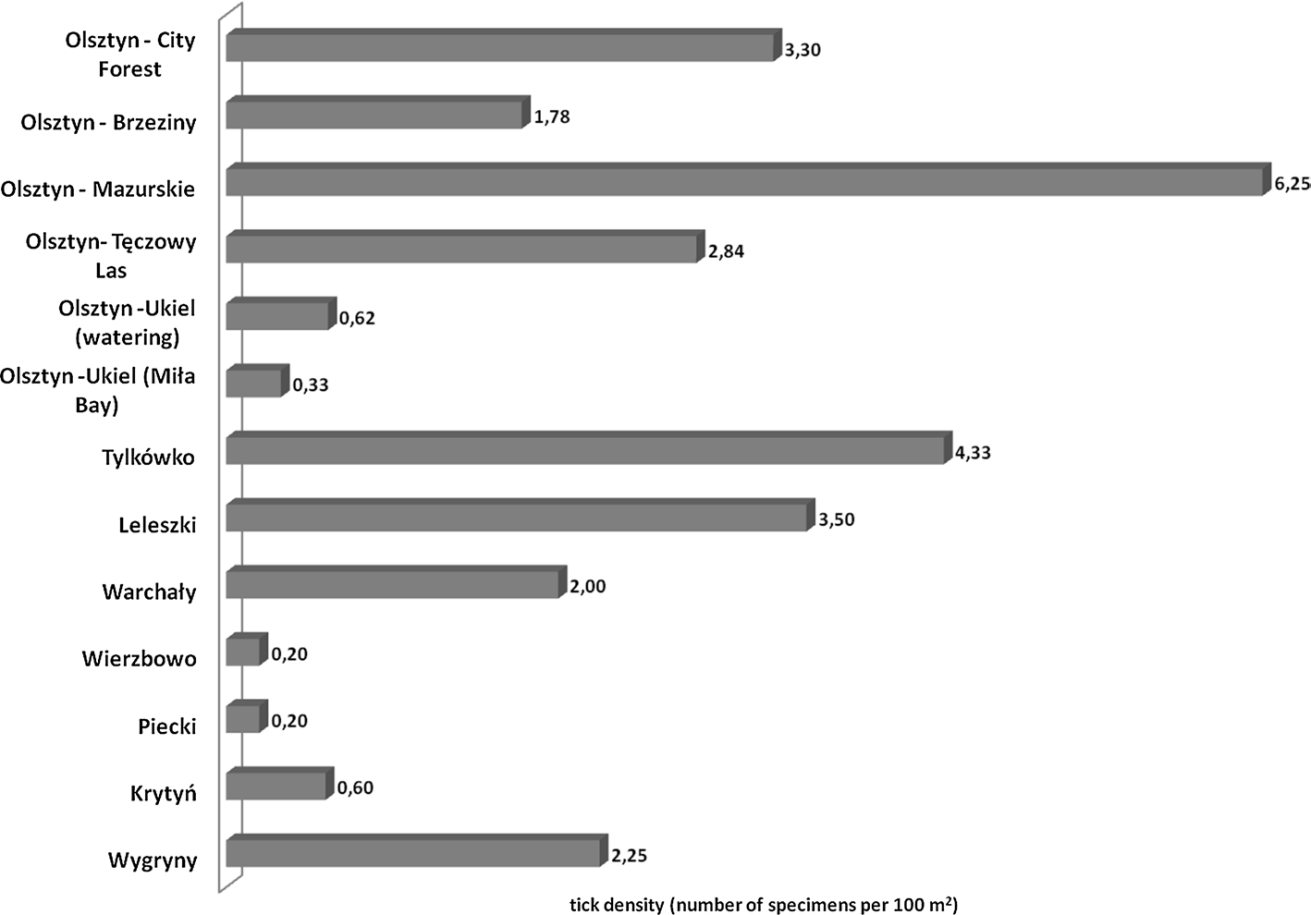
*Dermacentor reticulatus* density in the Warmia and Mazury region, north-eastern Poland (April, 2016)

During 2015–2017 a total of 398 adult *D. reticulatus* ticks were collected, including 257 females and 141 males (Table 1). In April 2016, depending on the locality, the density of ticks per 100 m^2^ ranged from 0.2 (Wierzbowo, Piecki) to 6.3 (Olsztyn–Mazurskie) (Fig. 2). Comparison of data grouped according to urban and natural type of area revealed no statistical differences between them, although the *D. reticulatus* tick density was slightly higher in an urban area (Table 2). Taking into account the habitat type, the mean tick density was the highest in open landscapes with 4.08 ticks per 100 m^2^. The *D. reticulatus* abundance in this habitat was significantly different only in comparison with the ecotone between forest and lake shore vegetation (Table 2).

**Table 2:** Mann–Whitney U test of differences in occurrence of* Dermacentor reticulatus* between habitats in north-eastern Poland (April, 2016)

Habitats	Mean density ± SD	Median (min–max) values	Mann–Whitney U	Asymptomatic significance (2-tailed)	Exact significance [2·(1-tailed significance)]
Urban	2.72 ± 2.615	2.60 (0.33–9.50)	30.0	0.441	0.479
Natural	1.87 ± 1.637	2.00 (0.20–4.33)			
Ecotone (grassy path/forest)	2.60 ± 1.383	2.60 (0.20–4.00)	12.0	0.371	0.432
Open landscape	4.08 ± 3.270	3.00 (0.89–9.50)			
Ecotone (grassy path/forest)	2.60 ± 1.383	2.60 (0.20–4.00)	5.0	0.088	0.109
Ecotone (forested areas and lake shore vegetation)	0.89 ± 0.754	0.61 (0.33–2.00)			
Ecotone (grassy path/forest)	2.60 ± 1.383	2.60 (0.20–4.00)	1.5	0.104	0.111
Forest landscapes	0.40 ± 0.283	0.40 (0.20–0.60)			
Open landscape	4.08 ± 3.270	3.00 (0.89–9.50)	1.0	0.027	0.032
Ecotone (forested areas and lake shore vegetation)	0.89 ± 0.754	0.61 (0.33–2.00)			
Open landscape	4.08 ± 3.270	3.00 (0.89–9.50)	0.0	0.053	0.095
Forest landscapes	0.40 ± 0.283	0.40 (0.20–0.60)			
Ecotone (forested areas and lake shore vegetation)	0.89 ± 0.754	0.61 (0.33–2.00)	2.0	0.926	0.533
Forest landscapes	0.40 ± 0.283	0.40 (0.20–0.60)			

## Discussion

The presented report supplements the data on the distribution of *D. reticulatus* in north-eastern Poland belonging to the endemic area for tick-borne diseases (TBDs). According to Vu Hai et al. (2014), one of the current tools for estimating the burden and risk of TBDs in Europe is an assessment of a tick’s distribution in relation to biotope, landscape, urbanization, climate and a description of its extension and potential risk area. The region of north-eastern Poland is considered typical for *D. reticulatus* and should be treated as a contiguous area of eastern populations of the meadow tick (Bogdaszewska 2004). However, to date the occurrence of *D. reticulatus* in this region has been recorded as separated foci of this tick species. In the province of Podlasie, the occurrence of *D. reticulatus* has been documented in the Augustów Primeval Forest (the Augustów focus), the Knyszyn Primeval Forest (the Knyszyn focus), the Biebrza Basin (the Biebrza focus) (Siuda 1993; Karbowiak 2014) and in the Białowieża National Park (Biernat et al. 2014) (Fig. 1). In Warmia and Mazury, *D. reticulatus* were found in the Mazury Lake District and Piska Primeval Forest (the Mazury focus) which covers the Pisz, Giżycko, Węgorzewo and Mrągowo districts (Siuda 1993; Bogdaszewska 2004; Karbowiak 2014; Mierzejewska et al. 2015a, b, c, 2016).

A 3-year-long monitoring of tick prevalence in north-eastern Poland demonstrated that *D. reticulatus* was permanently present in the central part of Warmia and Mazury (Olsztyn subregion), outside of the areas associated with the Mazury Lake District where, according to Bogdaszewska (2004), the reservoirs of this species and its transmission are impacted by increasing populations of roe deer and elk. Our observations have shown that in this area *D. reticulatus* is found both in natural biotopes and in habitats situated in a city, often close to housing complexes, and its density is comparable in both environments. Accordingly, Mierzejewska et al. (2015a) reported that the average density of *D. reticulatus* was higher in sites located near the capital city of Warsaw compared with the semi-natural biotopes typical of the Mazury Lake District. The adaptation of this tick species to the environmental conditions of large cities is evidenced by data on the host-seeking adult specimens on vegetation and domestic animals (mainly dogs) in other cities in Poland, such as Warsaw (Zygner 2006; Zygner et al. 2009), Wrocław (the west of Poland) (Kiewra and Czulowska 2013; Król et al. 2016), Lublin and Lubartów (the east of Poland) (Biaduń 2011) and also Kyiv (Didyk et al. 2017), Budapest (Földvári et al. 2016) and Košice (Bullová et al. 2009). In cities, *D. reticulatus* is usually found near large natural forest complexes surrounding the city, such as our locations: Olsztyn–Tęczowy Las, Olsztyn-City Forest or Olsztyn–Ukiel; or other locations in Warsaw (Supergan and Karbowiak 2009; Zygner et al. 2009). It makes the migration of mammals and other animals from natural forests to city green areas possible, along with any accompanying parasites and attached ticks (Karbowiak 2014). In urbanized areas, an important role can be played by birds, as they serve as hosts of the young stages of ticks. Other host may be companion animals, such as dogs or horses (Földvári et al. 2016). This was confirmed in the studies carried out by Mierzejewska et al. (2015c) who demonstrated that in *D. reticulatus* endemic areas in Poland, dogs and livestock (cows, horses) are their main hosts.

In urban areas, small city parks are usually unfavourable locations for ticks (Földvári et al. 2011; Rizzoli et al. 2014; Paulauskas et al. 2015), e.g. Lake Kortowskie in Olsztyn, where no specimens of this species were collected, although this location presents a typical habitat for *D. reticulatus*. Most probably, it results from the lack of large mammalian hosts and regular maintenance of greeneries (mowing, leaf raking). The ways in which the green areas that are *D. reticulatus* habitats are used significantly impacts a reduction in the density of this species (Mierzejewska et al. 2017). In the Warmia and Mazury region, meadows that were regularly harvested or post-fire sites have demonstrated a substantially lower density of *D. reticulatus* ticks in comparison with the adjacent undisturbed fallow lands. According to the author, it proves that an increase in the surface of fallows and changes in the methods of land usage (ceasing extensive land burning) will increase the number of habitats that are favourable for meadow ticks in Poland. This is further supported by the highly fragmented landscape within a large patch of homogeneous vegetation in proximity to permanent watercourses or reservoirs. The identified *D. reticulatus* habitats in north-eastern Poland confirm this relation. Most described habitats consist of untreated green wastelands that are often located at the border of forest and grass pathways or lakeside greeneries. In turn, the forest habitats, such as Krutyń, are situated near lakes and may be a part of game animal water tracks.

In north-eastern Poland, both in the urban areas and natural biotopes the presence of *D. reticulatus* ticks demonstrated in the study increases the risk of tick-borne diseases in humans and animals. It is thus warranted to monitor tick distribution and to determine the level of pathogen contamination in ticks in such areas, which is important from both medical and veterinary points of view.

## References

[CR1] Bartosik K, Wiśniowski Ł, Buczek A (2011). Abundance and seasonal activity of adult *Dermacentor reticulatus* (Acari: Amblyommidae) in eastern Poland in relation to meteorological conditions and the photoperiod. Ann Agric Environ Med.

[CR2] Biaduń W (2011). New habitats of *Dermacentor reticulatus* (Fabricius, 1794) in the Lublin region. Polish J Environ Stud.

[CR3] Biernat B, Karbowiak G, Werszko J, Stańczak J (2014). Prevalence of tick-borne encephalitis virus (TBEV) RNA in *Dermacentor reticulatus* ticks from natural and urban environment, Poland. Exp Appl Acarol.

[CR4] Bogdaszewska Z (2004). Występowanie i ekologia kleszcza łąkowego *Dermacentor reticulatus* (Fabricius, 1794) w ognisku mazurskim. I. Określenie obecnego zasięgu występowania (Range and ecology of *Dermacentor reticulatus* (Fabricius, 1794) in Mazuria focus. I. Defining current range). Wiadomości Parazytol.

[CR5] Bullová E, Lukáň M, Stanko M, Peťko B (2009). Spatial distribution of *Dermacentor reticulatus* tick in Slovakia in the beginning of the 21st century. Vet Parasitol.

[CR6] Chitimia-Dobler L (2015). Spatial distribution of *Dermacentor reticulatus* in Romania. Vet Parasitol.

[CR7] Dautel H, Dippel C, Oehme R (2006). Evidence for an increased geographical distribution of *Dermacentor reticulatus* in Germany and detection of *Rickettsia sp*. RpA4. Int J Med Microbiol.

[CR8] Didyk YM, Blaňárová L, Pogrebnyak S (2017). Emergence of tick-borne pathogens (*Borrelia burgdorferi* sensu lato*, Anaplasma phagocytophilum, Ricketsia raoultii* and *Babesia microti*) in the Kyiv urban parks, Ukraine. Ticks Tick Borne Dis.

[CR9] Földvári G, Rigó K, Jablonszky M (2011). Ticks and the city: Ectoparasites of the Northern white-breasted hedgehog (Erinaceus roumanicus) in an urban park. Ticks Tick Borne Dis.

[CR10] Földvári G, Široký P, Szekeres S (2016). *Dermacentor reticulatus*: a vector on the rise. Parasite Vectors.

[CR11] Jongejan F, Ringenier M, Putting M, Berger L (2015). Novel foci of *Dermacentor reticulatus* ticks infected with *Babesia canis* and *Babesia caballi* in the Netherlands and in Belgium. Parasite Vectors.

[CR12] Kadulski S, Izdebska J, Buczek A, Błaszak Cz (2009). New data on distribution of *Dermacentor reticulatus* (Fabr.) (Acari, Ixodidae) in Poland. Arthropods. Invasions their control.

[CR13] Karbowiak G (2014). The occurrence of the *Dermacentor reticulatus* tick: its expansion to new areas and possible causes. Ann Parasitol.

[CR14] Karbowiak G, Kiewra D (2010). New locations of *Dermacentor reticulatus* ticks in Western Poland: the first evidence of the merge in *D. reticulatus* occurrence areas?. Wiadomości Parazytol.

[CR15] Kiewra D, Czulowska A (2013). Evidence for an increased distribution range of *Dermacentor reticulatus* in south-west Poland. Exp Appl Acarol.

[CR16] Kloch A, Mierzejewska EJ, Karbowiak G (2017). Origins of recently emerged foci of the tick *Dermacentor reticulatus* in central Europe inferred from molecular markers. Vet Parasitol.

[CR17] Król N, Obiegala A, Pfeffer M (2016). Detection of selected pathogens in ticks collected from cats and dogs in the Wrocław Agglomeration, South-West Poland. Parasit Vectors.

[CR18] Kubiak K, Sielawa H, Tylkowska A et al (2017) Prevalence of tick-borne pathogens in questing *Dermacentor reticulatus* (Fabr. 1794) ticks in north-eastern Poland. In: The XVI conference of Ukrainian scientific society of parasitologists (abstracts), 18–21 September, 2017, Lviv, Ukraina, p 103

[CR19] Mierzejewska EJ, Alsarraf M, Behnke JM, Bajer A (2015). The effect of changes in agricultural practices on the density of *Dermacentor reticulatus* ticks. Vet Parasitol.

[CR20] Mierzejewska EJ, Pawełczyk A, Radkowski M (2015). Pathogens vectored by the tick, *Dermacentor reticulatus*, in endemic regions and zones of expansion in Poland. Parasit Vectors.

[CR21] Mierzejewska EJ, Welc-Falęciak R, Karbowiak G (2015). Dominance of *Dermacentor reticulatus* over *Ixodes ricinus* (Ixodidae) on livestock, companion animals and wild ruminants in eastern and central Poland. Exp Appl Acarol.

[CR22] Mierzejewska EJ, Estrada-Peña A, Alsarraf M (2016). Mapping of *Dermacentor reticulatus* expansion in Poland in 2012–2014. Ticks Tick Borne Dis.

[CR23] Mierzejewska EJ, Estrada-Peña A, Bajer A (2017). Spread of *Dermacentor reticulatus* is associated with the loss of forest area. Exp Appl Acarol.

[CR24] NIH-PZH Infectious diseases and poisonings in Poland in 2013–2016. Natl Inst Public Heal-Natl Inst Hyg-Dep Epidemiol http://www.pzh.gov.pl/oldpage/epimeld/index_p.htm

[CR25] Nowak M (2011). Discovery of *Dermacentor reticulatus* (Acari: Amblyommidae) populations in the Lubuskie Province (Western Poland). Exp Appl Acarol.

[CR26] Nowak-Chmura M (2013). Fauna kleszczy (Ixodida) Europy Środkowej.

[CR27] Paulauskas A, Radzijevskaja J, Mardosaite-Busaitiene D (2015). New localities of *Dermacentor reticulatus* ticks in the Baltic countries. Ticks Tick Borne Dis.

[CR28] Reye AL, Stegniy V, Mishaeva NP (2013). Prevalence of tick-borne pathogens in *Ixodes ricinus* and *Dermacentor reticulatus* ticks from different geographical locations in Belarus. PLoS ONE.

[CR29] Rizzoli A, Silaghi C, Obiegala A (2014). *Ixodes ricinus* and its transmitted pathogens in urban and peri-urban areas in Europe: new hazards and relevance for public health. Front Public Heal.

[CR30] Rubel F, Brugger K, Pfeffer M (2016). Geographical distribution of *Dermacentor marginatus* and *Dermacentor reticulatus* in Europe. Ticks Tick Borne Dis.

[CR31] Široký P, Kubelová M, Bednář M (2011). The distribution and spreading pattern of *Dermacentor reticulatus* over its threshold area in the Czech Republic: How much is range of this vector expanding?. Vet Parasitol.

[CR32] Siuda K (1993) Kleszcze Polski (Acari: Ixodida). Systematyka i rozmieszczenie część II. (Ticks (Acari: Ixodida) of Poland. Part II Taxonomy and Distribution) Polskie Towarzystwo Parazytologiczne, Warszawa

[CR33] Supergan M, Karbowiak G (2009). The estimation scale of endangerment with tick attacks on recreational towns areas. Prz Epidemiol.

[CR34] Szymański S (1986). Distribution of the tick *Dermacentor reticulatus* (Fabricius, 1794) (Ixodidae) in Poland. Acta Parasitol Pol.

[CR35] Vu Hai V, Almeras L, Socolovschi C (2014). Monitoring human tick-borne disease risk and tick bite exposure in Europe: available tools and promising future methods. Ticks Tick Borne Dis.

[CR36] Zając V, Wójcik-Fatla A, Sawczyn A (2017). Prevalence of infections and co-infections with 6 pathogens in *Dermacentor reticulatus* ticks collected in eastern Poland. Ann Agric Environ Med.

[CR37] Zygner W (2006). Occurence of hard ticks in dogs from Warsow area. Ann Agric Environ Med.

[CR38] Zygner W, Górski P, Wçdrychowicz H (2009). New localities of *Dermacentor reticulatus* tick (vector of *Babesia canis canis*) in central and eastern Poland. Pol J Vet Sci.

